# A novel nomogram and risk stratification for early metastasis in cervical cancer after radical radiotherapy

**DOI:** 10.1002/cam4.6745

**Published:** 2023-11-23

**Authors:** Linying Liu, Jie Lin, Sufang Deng, Haijuan Yu, Ning Xie, Yang Sun

**Affiliations:** ^1^ Department of Gynecology Clinical Oncology School of Fujian Medical University, Fujian Cancer Hospital Fuzhou China

**Keywords:** cervical cancer, early distant metastasis, prognostic model, radiotherapy, risk factors

## Abstract

**Object:**

This study aimed to establish an effective risk nomogram to predict the early distant metastasis (EDM) probability of cervical cancer (CC) patients treated with radical radiotherapy to aid individualized clinical decision‐making.

**Methods:**

A total of 489 patients with biopsy‐confirmed CC between December 2018 and January 2021 were enrolled. Logistic regression with the stepwise backward method was used to identify independent risk factors. The nomogram efficacy was evaluated by using the area under the receiver operating characteristic curve (AUC), C‐index by 1000 bootstrap replications, etc. Finally, patients were divided into high‐ and low‐risk groups of EDM based on the cut‐off value of nomogram points.

**Results:**

36 (7.36%) CC patients had EDM, and 20 (55.6%) EDM had more than one metastatic site involved. Age below 51 (OR = 2.298, *p* < 0.001), tumor size larger than 4.5 cm (OR = 3.817, *p* < 0.001) and radiotherapy (OR = 3.319, *p* < 0.001) were independent risk factors of EDM. For the nomogram model, C‐index was 0.701 (95% CI = 0.604–0.798), and 0.675 (95% CI = 0.578–0.760) after 1000 bootstrap resampling validations. The Hosmer–Lemeshow test demonstrated no overfitting (*p* = 0.924). According to the Kaplan–Meier curve of risk score, patients with high risk were more prone to get EDM (*p* < 0.001).

**Conclusion:**

This is the first research to focus on EDM in CC patients. We have developed a robust scoring system to predict the risk of EDM in CC patients to screen out appropriate cases for consolidation therapy and more intensive follow‐up.

## 1 INTRODUCTION

Cervical cancer (CC) ranks as the fourth most common female malignancy worldwide with a significant portion of the global cancer burdens for women.^1^, ^2^ It was estimated that there will be 604,127 new cases and 341,831 deaths related to CC in 2022 worldwide.^3^ Cervical cancer is prevalent in low‐ and middle‐income countries, especially in China.^2^, ^4^ Radiation therapy (RT) is recommended as an optional primary therapy for CC patients at all stages by the National Comprehensive Cancer Network (NCCN) guidelines. However, it has limitations in preventing distant metastasis (DM).^5^ More than 20% of CC patients suffered from DM^5^, ^6^, ^7^ and the 5‐year survival rate was only 17%.^8^, ^9^ Distant metastasis is now the main treatment failure pattern of CC.^8^, ^9^ Even with the application of small molecular agents like bevacizumab or immunotherapy such as pembrolizumab, the survival outcomes are not satisfying while creating financial burdens simultaneously.^6^, ^10^ Therefore, it is urgent to predict the susceptibilities of DM in CC patients to make personalized treatment plans and alleviate such dilemmas.

Distant metastasis is defined as an original primary tumor developing into a distal secondary tumor.^11^ There have been several studies conducted to determine the attributes of DM in CC.^5^, ^7^ It is mentionable that some cancer patients who have finished complete treatments experience DM quickly, which could not be anticipated well. This kind of metastasis has recently been the hot spot in cancer research.^12^, ^13^, ^14^ Compared to late DM, patients with early distant metastases (EDM) have a poorer prognosis due to the compliance and tolerance reduction of secondary treatment.^13^, ^15^ What's worse, patients are always inert to the second radiotherapy or chemotherapy.^11^ In clinical practice, adding systematic and intensive treatments prior could reduce EDM possibilities and improve survival outcomes.^16^, ^17^ In the well‐known randomized phase III OUTBACK trial, consolidation chemotherapy could not bring benefit for local advanced cervical cancer with standard concurrent chemoradiotherapy.^18^, ^19^ However, risk factors of distant metastasis like larger tumor size, lymph nodes metastasis situation, and their proportion were not described. Identifying high‐risk group who would develop EDM was meaningful to help to find out patients who could benefit from consolidation therapy. To our concern, EDM has not been highlighted in CC. There are also few reliable models to predict EDM susceptibility in CC patients. Our study aims to identify attributive risk factors of EDM to help optimize personalized treatment plans and even to prevent metastasis in CC patients with radical RT.

## 2 METHODS AND MATERIALS

### 2.1 Study population

Our study was approved by the Institutional Review Board of the Fujian Cancer Hospital Ethics Committee, in compliance with the Declaration of Helsinki (SQ2021‐017‐01).

From December 2018 to January 2021, 1529 patients confirmed to have CC by biopsies and treated with RT were initially enrolled in Fujian Cancer Hospital. Pre‐treatment evaluation included patient history, physical (gynecological) examinations, blood analysis, thoracic, abdominal and pelvic CT, or whole body FDG‐PET‐CT and pelvic MRI. All patients were staged based on the FIGO staging system (2018 version). The 1040 patients were excluded according to the criteria as follows: (1) patients treated with CC surgery. (2) Patients received incomplete CC treatment somewhere else. (3) Patients with cancer history. (4) Patients with pelvic surgery history. (5) Patients without complete pre‐treatment medical information. (6) Patients with unfinished radical radiotherapy. (7) Patients suffered from DM within 3 months after radiotherapy. (8) Patients without complete 1‐year follow‐up materials. Finally, 489 CC patients were included.

### 2.2 Data collection

The baseline parameters involved age, treatment, RT dosage, pathology, stage, tumor size, pelvic lymph node metastasis (PLNM), para‐aortic lymph node metastasis (PALNM), inflammatory index, nutritional indexes, hemoglobin (HB) and albumin (ALB). The blood biochemistry data were collected within 5 days before therapy.

#### 2.2.1 Inflammatory and nutritional index

The inflammatory and nutritional indexes were calculated using the blood biochemical data. The systemic immune‐inflammation index (SII) = absolute neutrophil count × absolute platelet count ÷ absolute lymphocyte count. Neutrophil–lymphocyte ratio (NLR) = absolute neutrophil count ÷ absolute lymphocyte count. Platelet–lymphocyte ratio (PLR) = absolute platelet count ÷ absolute lymphocyte count. Platelet–albumin ratio (PAR) = absolute platelet count ÷ serum albumin level. Prognostic‐nutrition index (PNI) = serum albumin level + 5 × the absolute lymphocyte count.

### 2.3 Treatment strategies

#### 2.3.1 Radical RT

Radical RT consisted of external pelvic beam radiotherapy (EBRT) followed by individualized high‐dose‐rate intracavitary brachytherapy (HDR‐ICBT) with 192 Ir. EBRT was performed with intensity‐modulated radiotherapy (IMRT) or conventional 4 or 6‐field box conformal RT technique. The external whole‐pelvis irradiation was delivered at 1.8–2.0 Gy per fraction for five fractions per week up to a total external dose of 45.0–50.0 Gy. If common iliac lymph nodes were positive, para‐aortic lymph nodes would also be prophylactically irradiated with a dose of 45.0–50.0 Gy. The para‐aortic radiation field was up to the level of renal vessels or even more cephalad and directed by involved nodal distribution. The RT dose was boosted for positive pelvic or para‐aortic lymph nodes to 10–16 Gy. After adequate tumor regression, HDR‐ICBT was performed with a fractional dose of 7.0 Gy per week to 28.0 Gy in 4 weeks. The overall dose should be more than 75 Gy (EQD2).

#### 2.3.2 Chemotherapy

Cisplatin‐based concurrent chemoradiotherapy (CCRT) was recommended for CC patients according to the guideline of NCCN. Cisplatin (40 mg/m^2^) or nedaplatin (80 mg/m^2^) monotherapy or combined with paclitaxel (135 mg/m^2^) were administered intravenously every 3 weeks during pelvic RT. However, some patients were treated with RT alone due to their preferences, economic status, and age consideration.

### 2.4 Follow‐up evaluations and outcomes

After treatment, patients were advised to be reexamined every 3 months in the first 2 years, every 6 months in the 3–5 years, and once a year after 5 years. While this follow‐up schedule was not permanently fixed. Follow‐up examination consisted of clinical history, physical (gynecological) examinations, blood analysis, thoracic and abdominal CT, and pelvic MRI or CT. When any signs of DM or recurrence were observed, supplementary examinations such as whole‐body bone scan, PET/CT, or histopathologic examination were performed.

#### 2.4.1 The definition of EDM

The primary endpoint was EDM referring to any disease relapses out of the radiation field within a short period after therapy. Considering persistent disease occurs in three to 6 months after radiotherapy completion,^20^ we defined EDM as metastasis occurrence between 3 and 12 months after RT.

### 2.5 Statistical analyses

Statistical analyses were performed using R software version 4.2.2 (http://www.r‐project.org/). Categorical variables like stage, treatment, pathology, etc. were classified based on clinical findings. Continuous variables were transformed into categorical variables based on cut‐off points using the X‐tile software version 3.6.1, which is essential for generating the best cut‐off point with the minimum p‐value. Firstly, the variables that exhibited an original significance level of *p* < 0.10 in the univariate analysis were entered into multivariate analyses. A backward stepwise method was used for variable selection in binary logistic regression. Then, the nomograms were created using the ‘rms’ package. And the area under the curves (AUC) was calculated to evaluate the performance of the predictive model. Next, calibration plots and C‐indices were generated to test the predictive accuracy using 1000 bootstrap resamples to decrease the overfitting bias. Furthermore, decision curve analysis (DCA) was performed to illustrate the accuracy of the model by calculating the net benefit over a spectrum of probability thresholds. Finally, the score of each predictor was assigned according to the points of the nomogram correspondingly. And patients were divided into high‐ and low‐risk groups based on the cut‐off value of the risk score calculated by summing up each risk factor. Early distant metastasis probability was observed by the Kaplan–Meier analysis. The *p*‐value < 0.05 was considered to be statistically significant.

## 3 RESULTS

### 3.1 Baseline characteristics and clinical outcomes

**FIGURE 1:**
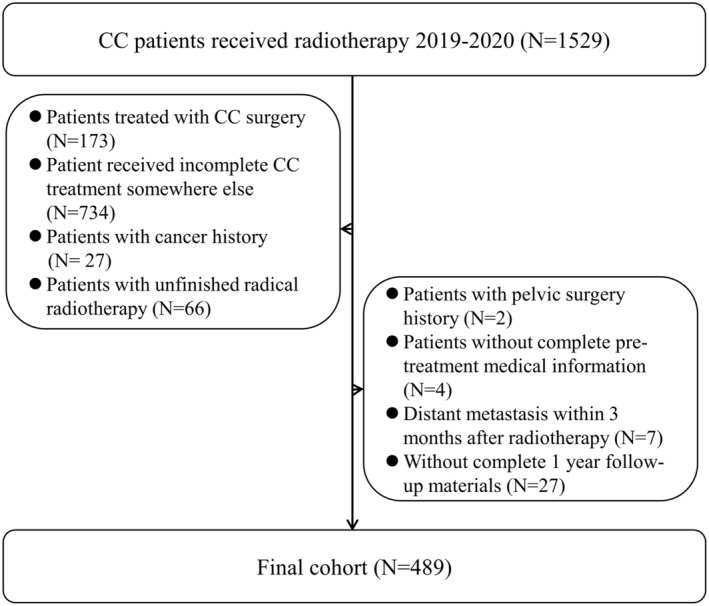
Flowchart of patient eligibility screening.

Figure 1 shows the flowchart of this study, and 489 were enrolled finally. The follow‐up continued until February 2023. The median follow‐up time was 33.1 months (range: 3.5–48.9 months). The baseline characteristics are summarized in Table 1. 36 (7.4%) CC patients developed EDM. Compared to non‐early distant metastasis (NEDM) patients, patients who experienced EDM were more likely to have larger tumor sizes (72.2% vs. 47%, *p* < 0.05) and prone to be treated with RT only (36.1% vs. 19.9%, *p* < 0.05). While the left features had no significant difference. Early distant metastasis details are shown in Table 2. The most common EDM sites were lungs (*n* = 19), followed by lymph node regions out of the field (*n* = 17). Particularly, 20 (55.6%) patients with EDM had more than one region or organ metastasis.

**TABLE 1:** Clinicopathologic characteristics of participants.

Items	NEDM	EDM	*p*
	*n* = 453	*n* = 36	
Age (years)			0.077
≤51	120 (26.5%)	15 (41.7%)	
>51	333 (73.5%)	21 (58.3%)	
Treatment			0.037
CCRT	363 (80.1%)	23 (63.9%)	
RT	90 (19.9%)	13 (36.1%)	
Total dose			0.39
≤87.3 Gy	191 (42.2%)	12 (33.3%)	
>87.3 Gy	262 (57.8%)	24 (66.7%)	
Pathology			0.252
Squamous‐cell carcinoma	430 (94.9%)	32 (88.9%)	
Non‐squamous‐cell carcinoma	23 (5.1%)	4 (11.1%)	
Stage			0.754
IB‐IIIB	320 (70.6%)	24 (66.7%)	
IIIC‐IVA	133 (29.4%)	12 (33.3%)	
PLNM			0.545
Non‐PLNM	330 (72.8%)	24 (66.7%)	
PLNM	123 (27.2%)	12 (33.3%)	
PALNM			0.981
Non‐PALNM	435 (96%)	34 (94.4%)	
PALNM	18 (4%)	2 (5.6%)	
Tumor size			0.006
≤4.5 cm	240 (53%)	10 (27.8%)	
>4.5 cm	213 (47%)	26 (72.2%)	
SII			0.226
≤788.4	301 (66.4%)	28 (77.8%)	
>788.4	152 (33.6%)	8 (22.2%)	
NLR			1
≤1.6	107 (23.6%)	8 (22.2%)	
>1.6	346 (76.4%)	28 (77.8%)	
PLR			0.214
≤94.7	50 (11%)	7 (19.4%)	
>94.7	403 (89%)	29 (80.6%)	
PNI			0.272
≤45.3	53 (11.7%)	7 (19.4%)	
>45.3	400 (88.3%)	29 (80.6%)	
PAR			0.27
≤5	74 (16.3%)	9 (25%)	
>5	379 (83.7%)	27 (75%)	
HB			0.175
≤132	323 (71.3%)	30 (83.3%)	
>132	130 (28.7%)	6 (16.7%)	
ALB			0.314
≤37.2	55 (12.1%)	7 (19.4%)	
>37.2	398 (87.9%)	29 (80.6%)	

**TABLE 2:** Patterns of EDM.

Patterns of EDM	Total number
Lung	19
Lymph node	17
Supraclavicular lymph node	11
Other	6
Bone	7
Multiple sites	20

### 3.2 Identification of predictors for EDM

The univariable and multivariable logistic analyses of EDM are shown in Table 3. The age (<51 years old) (OR = 2.42, 95% CI = 1.12–5.20, *p* = 0.025), tumor size (>4.5 cm) (OR = 2.85, 95% CI = 1.32–6.13, *p* = 0.008) and RT (OR = 3.29, 95% CI = 1.49–7.30, *p* = 0.003) were independent risk factors for EDM.

**TABLE 3:** Univariate and multivariate logistic analyses regarding EDM for CC patients.

Items	Univariate		Multivariate	
	OR (95% CI)	*p*	OR (95% CI)	*p*
Age ≤ 51	1.98 (0.99–3.97)	0.054	2.41 (1.12–5.20)	0.025
RT	2.28 (1.11–4.67)	0.024	3.29 (1.49–7.30)	0.003
Total dose >87.3 Gy	1.46 (0.71–2.99)	0.303		
Non‐squamous‐cell carcinoma	2.34 (0.76–7.17)	0.138		
Stage IIIC‐IVA	1.20 (0.58–2.48)	0.616		
Pelvic lymph node metastasis	1.34 (0.65–2.76)	0.426		
Para‐aortic lymph node metastasis	1.42 (0.32–6.38)	0.646		
Tumor size >4.5 cm	2.93 (1.38–6.22)	0.005	2.85 (1.32–6.13)	0.008
SII > 788.4	0.57 (0.25–1.27)	0.168		
NLR > 1.6	1.08 (0.48–2.45)	0.849		
PLR > 94.7	0.51 (0.21–1.2)	0.137		
PNI > 45.3	0.55 (0.23–1.32)	0.178		
PAR > 5	0.59 (0.26–1.30)	0.187		
HB > 132	0.50 (0.20–1.22)	0.128		
ALB > 37.2	0.57 (0.24–1.37)	0.21		

### 3.3 Development and validation of a nomogram

**FIGURE 2:**
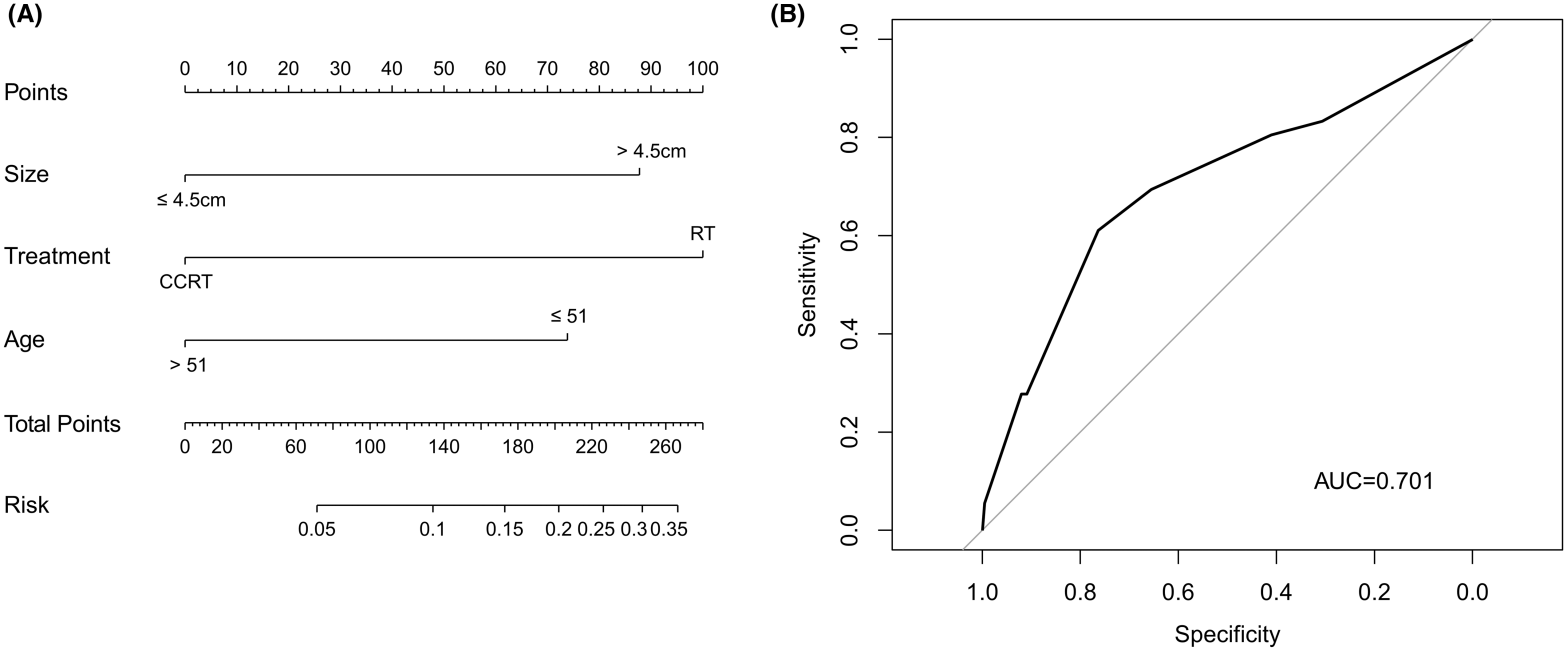
Nomogram (A) and Receiver operating characteristic (ROC) curve (B) for predicting the probability of EDM.

**FIGURE 3:**
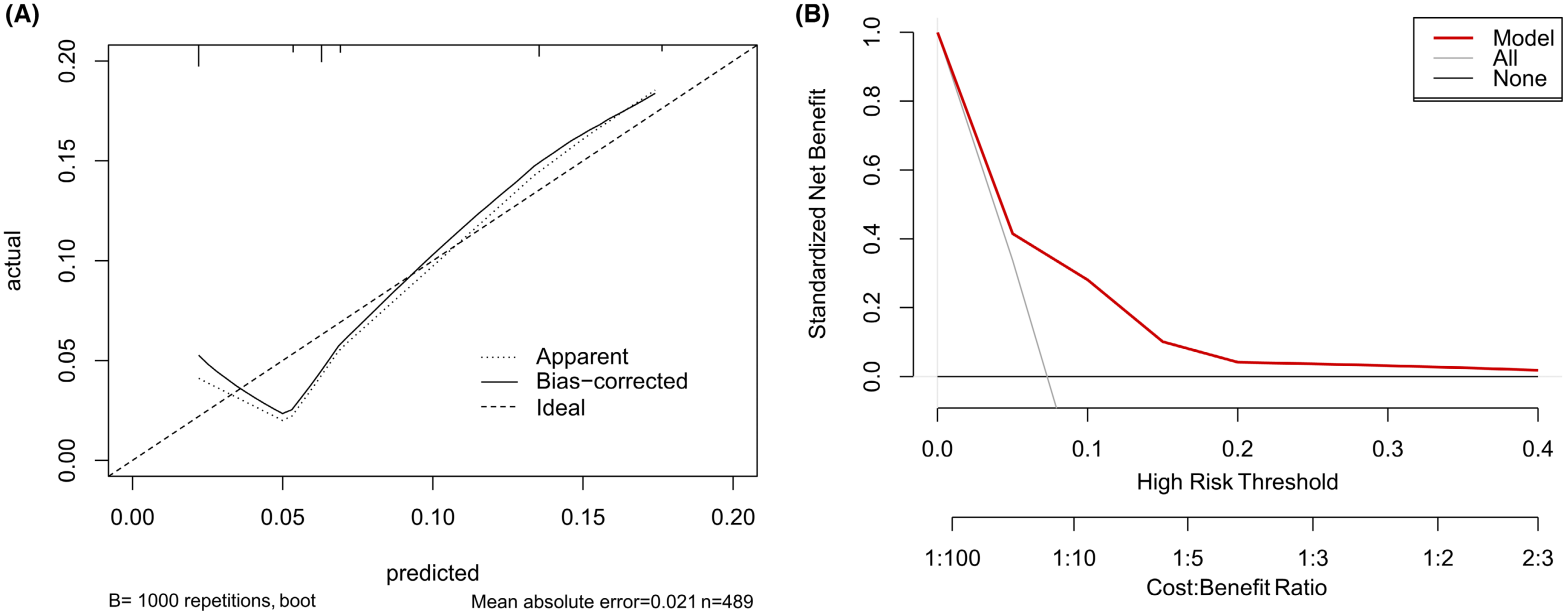
Calibration plots (A) and DCA (B) of the nomogram predicting EDM.

Age, tumor size, and RT were selected to formulate a nomogram (Figure 2A). The AUC for the combination of these three predictors was 0.701 (95% CI = 0.604–0.798) (Figure 2B). The accuracy of this prediction model was relatively convincing. The C‐index for the prediction model was 0.701 (95% CI = 0.604–0.798) and was corrected to 0.675 (95% CI = 0.578–0.760) by bootstrap 1000 resampling validation, which suggested a good discriminatory ability. A calibration curve showed good consistency between the actual and predicted EDM probability (Figure 3A). The bias‐corrected calibration plot showed a limited departure from the ideal line with a mean absolute error of 2.1%, and the Hosmer–Lemeshow test demonstrated no overfitting (*p* = 0.924). Additionally, the DCA showed that the nomogram can bring net positive benefit when the threshold probability ranged from 0.02 to 0.4 (Figure 3B).

### 3.4 Establishment and simplification of the EDM scoring system

**FIGURE 4:**
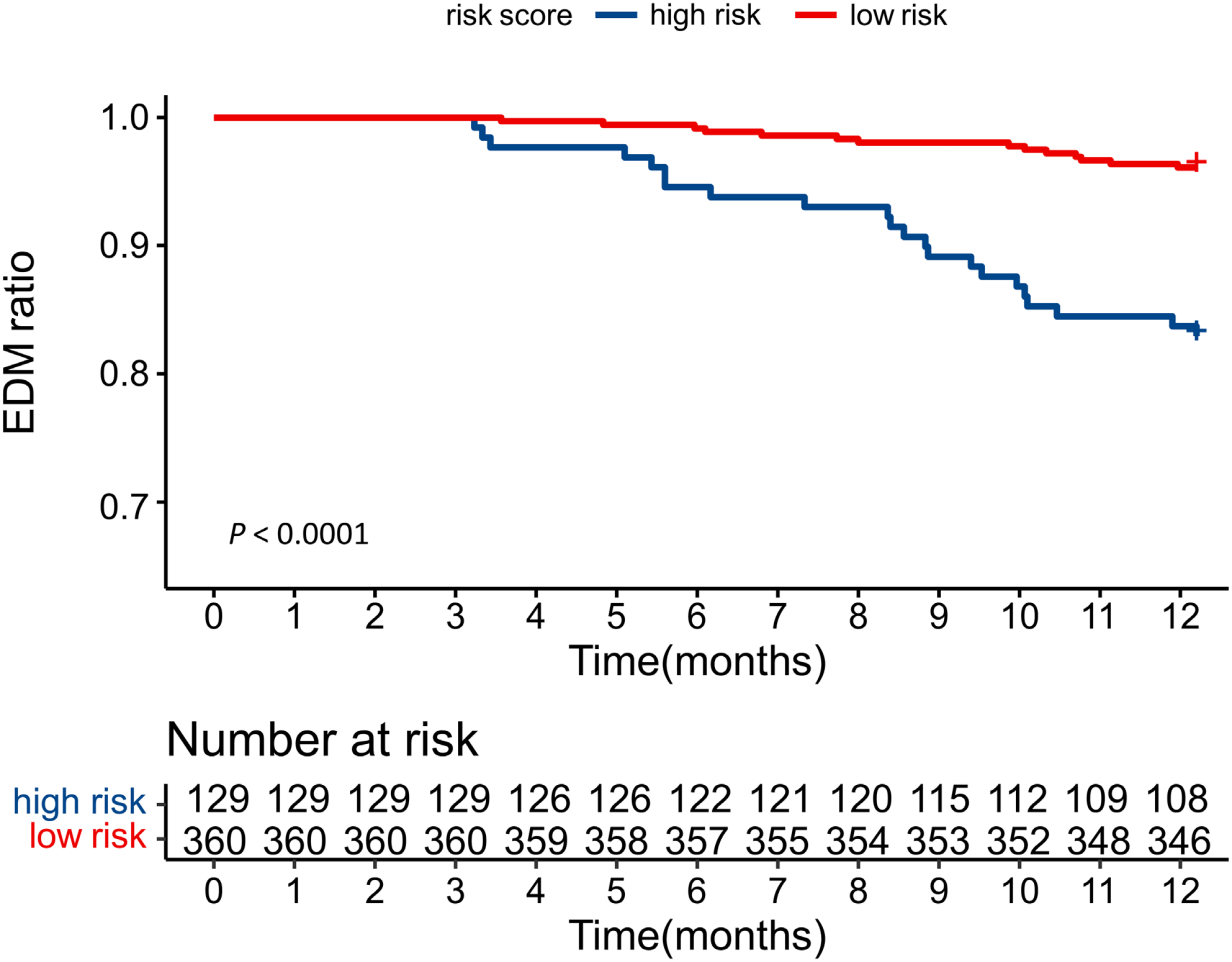
Kaplan–Meier analysis in high‐and low‐ risk groups.

For clinical use, a scoring system was built based on the nomogram. By drawing vertical lines on different fraction axes of each covariate, the individual risk factor points were assigned respectively (age (≤51 years old): 73.8, tumor size (>4.5 cm): 87.8, and RT: 100), and the referent for each variable was assigned 0. The total scores ranged from 0 to 261.6 by summing up those factors. According to the optimal value of total scores (cutoff = 161.6), 360 patients were stratified into low‐risk group and 129 patients in high‐risk groups. To be more specific, patients with none or only one risk factor were allocated into low‐risk groups, and others with more than two factors were in high‐risk group. The cumulative probability of the occurrence of EDM was significantly higher in the high‐risk group (*p* < 0.0001) (Figure 4).

## 4 DISCUSSION

With the implementation of modern conformal RT techniques and the optimization of the local control rate, DM has become an urgent issue leading to a dismal prognosis in CC.^16^, ^21^ As a vital portion of DM, EDM will lead to a worse prognosis, but it has not been attached to great importance in CC. In this study, the age (≤51 years old), tumor size (>4.5 cm), and RT were identified as independent risk factors for EDM, which have been proven to be survival predictors of CC.^22^ Our predictive nomogram integrated with these three factors showed good predictive performance with good discrimination and calibration. To the best of our knowledge, this is the first study to screen CC patients with EDM and the first nomogram to predict the risk of EDM.

The EDM of CC showed more aggressive characteristics and led to worse outcomes. The EDM rate of 7.4% in our study was nearly half of the 5‐year DM rate reported, which revealed the significance of the EDM portion in DM.^7^ Additionally, as the main EDM type, hematogenous metastasis indicated adverse outcomes. In this study, lung was the main metastatic site exceeding lymph node region. Interestingly, instead of hematogenous metastasis, lymph node metastasis (LNM) was the main pattern of DM in previous studies.^5^ It has been demonstrated that CC patients with hematogenous metastasis had a 5.3‐fold higher risk of death compared to those with lymphatic metastasis,^23^ and almost half of the hematogenous metastasis CC patients would die within 6 months.^11^ Furthermore, over 40% of EDM involved multi‐site relapse. As chemotherapy had a limited influence on survival outcomes once DM occurred,^11^ treatment difficulties would increase without favorable therapies within a short time. EDM plays a significant role in exacerbating the prognosis of CC patients. Therefore, it's meaningful to explore risk contributors for patients with EDM.

Our study incorporated tumor size (>4.5 cm) as one of the EDM predictors. Several studies have proven that tumor size larger than 4 cm was a predictor of increasing DM occurrence or treatment failure in CC patients.^24^, ^25^ A commonly accepted theory was that as cancer grew, tumor cells acquired the capability to spread, survive, and flourish.^26^ Furthermore, tumor size has proven to be associated with risk factors of DM occurrence including lymph‐vascular space invasion,^27^ parametrial involvement,^28^, ^29^ and hypoxic volume.^30^, ^31^ Additionally, tumor size had a time‐varying effect on recurrence.^32^ Chung Chang found that CC patients with larger tumor size were faced with a higher risk for recurrence in the first 10 months after initial treatment. Hence, it should pay attention to the role of tumor size plays in EDM development.

Age (≤51 years old) was also identified as an EDM predictor. Many studies showed elder CC patients were prone to receive more conservative treatments including smaller EBRT field and less radiotherapy dose, which led to similar DM rates between young and elder groups.^33^, ^34^ In our study, elder patients received complete radical therapy. We presumed the more aggressive treatments for elder patients were the reasons for the EDM rates improvement in the elderly and finally result in higher risk of EDM at a young age.^33^ What's more, age might negatively correlate with radioresistance.^35^ A large epidemiologic and clinical analysis in Japan showed that the prognosis at a young age was poorer with radiation‐based treatment. Young patients with radioresistance had unfavored local control rates, which might also result in EDM susceptibility.^36^ More research is needed to further explain.

Besides two pretreatment risk factors, RT showed great importance in EDM. EDM occurrence might be closely related to the elimination of primary tumors and residual lesions. Chemotherapy as a systemic therapy work against unseen residual lesion and micrometastasis.^37^ M. P. Schmid et al.^38^ revealed that the chemotherapy cycles had significant impacts on DM occurrence in CC patients with advanced stages and LNM. A full 5–6‐cycle chemotherapy had the lowest DM rates followed by 1–4 cycles and without chemotherapy. In our study, we found that chemotherapy will reduce the risk of EDM regardless of the terms of cycles. Our findings proved and supplemented the significance of chemotherapy used in short‐ and long‐term prevention of DM. According to our results, we suggest young patients with smaller tumor size and elder patients regardless of tumor size should receive at least one cycle of chemotherapy to prevent EDM. While, for patients with young age and larger tumor size, not only chemotherapy but also an intensive and comprehensive follow‐up plan was recommended. Other systemic treatments including immunotherapy and targeted therapy have promising prospects and advantages in high‐risk patients.

The left factors in our study including pathology, inflammatory index, nutrition state, HB, and LNM were proven to be influential for CC patient prognosis.^5^, ^7^, ^38^, ^39^, ^40^, ^41^ The relationships between those factors and rapid progression have not been investigated, which was supplemented in our study. Although it appeared that these factors listed did not show significant predictive values in EDM occurrence, the discrepancy might be explained by a larger sample size cohort and further studies.

There were merits of this study. Firstly, we developed a nomogram model using rapidly available predictors including age, treatment, and tumor size, which help clinicians to predict EDM occurrence when treatment started. Additionally, a scoring system was calculated to help clinicians determine subsequent treatment and follow‐up schedules before CC patients received radical radiotherapy. Finally, to the best of our knowledge, this is the first study that focuses on the CC patients who experience EDM after radical radiotherapy and the first nomogram to predict the risk of EDM. However, there were also several limitations in our study. Firstly, selection bias was unavoidable as a retrospective study. Secondly, the EDM cases were relatively small, a larger population study could be further investigated in future research. Thirdly, the study result requires multicenter data to further validate its accuracy and clinical applicability. Finally, some known prognostic factors of CC, such as tumor markers, human papillomavirus (HPV) infections, LNM details and chemotherapy details were not incorporated into the study.

## 5 CONCLUSION

Age, tumor size, and RT were independent risk factors for the development of EDM in CC patients treated with RT. A practical nomogram model based on converting the combination of these three predictors had good translatability and discriminatory abilities. Based on our scoring system, consolidation therapies and more intensive follow‐up strategies were necessary for the CC patients at high risk for EDM status.

## AUTHOR CONTRIBUTIONS

**Linying Liu:** Formal analysis (equal); software (equal); writing – original draft (equal). **Jie Lin:** Formal analysis (equal); methodology (equal); writing – review and editing (equal). **Sufang Deng:** Conceptualization (equal); formal analysis (equal); software (equal). **Haijuan Yu:** Conceptualization (equal); formal analysis (equal). **Ning Xie:** Formal analysis (equal). **Yang Sun:** Funding acquisition (equal); project administration (equal); supervision (equal).

## FUNDING INFORMATION

This work was sponsored by the Major Project for Young and Middle‐aged Health Researchers of Fujian Province, China (Grant number: 2022ZQNZD008).

## CONFLICT OF INTEREST STATEMENT

The authors declare that they have no competing interests.

## ETHICS STATEMENT

This study was approved by the medical ethical committee review board of the Fujian Cancer Hospital (SQ2021‐017‐01) and was in accordance with the 1964 Helsinki declaration and its later amendments or comparable ethical standards. Patient identifiers such as names were not collected, instead patients were given a numerical identifier. For confidentiality, the patients' charts were used only within the confines of the records department and only the investigators and study assistant had access to the files.

## CONSENT FOR PUBLICATION

Approval for this retrospective observational study was obtained from the hospital research ethics board, and the requirement to obtain informed consent was waived.

## Data Availability

The data used or analyzed of the current study are available from the corresponding author on reasonable request.
